# Growth Factor Deregulation and Emerging Role of Phosphatases in Diabetic Peripheral Artery Disease

**DOI:** 10.3389/fcvm.2020.619612

**Published:** 2021-01-07

**Authors:** Clément Mercier, Marina Rousseau, Pedro Geraldes

**Affiliations:** Department of Medicine, Division of Endocrinology, Research Center of the Centre Hospitalier Universitaire de Sherbrooke, Université de Sherbrooke, Sherbrooke, QC, Canada

**Keywords:** peripheral arterial disease, diabetes, growth factor, phosphatase, endothelia cell dysfunction

## Abstract

Peripheral artery disease is caused by atherosclerosis of lower extremity arteries leading to the loss of blood perfusion and subsequent critical ischemia. The presence of diabetes mellitus is an important risk factor that greatly increases the incidence, the progression and the severity of the disease. In addition to accelerated disease progression, diabetic patients are also more susceptible to develop serious impairment of their walking abilities through an increased risk of lower limb amputation. Hyperglycemia is known to alter the physiological development of collateral arteries in response to ischemia. Deregulation in the production of several critical pro-angiogenic factors has been reported in diabetes along with vascular cell unresponsiveness in initiating angiogenic processes. Among the multiple molecular mechanisms involved in the angiogenic response, protein tyrosine phosphatases are potent regulators by dephosphorylating pro-angiogenic tyrosine kinase receptors. However, evidence has indicated that diabetes-induced deregulation of phosphatases contributes to the progression of several micro and macrovascular complications. This review provides an overview of growth factor alterations in the context of diabetes and peripheral artery disease, as well as a description of the role of phosphatases in the regulation of angiogenic pathways followed by an analysis of the effects of hyperglycemia on the modulation of protein tyrosine phosphatase expression and activity. Knowledge of the role of phosphatases in diabetic peripheral artery disease will help the development of future therapeutics to locally regulate phosphatases and improve angiogenesis.

## Methods of Bibliographic Search

The bibliographic search was performed in the PubMed and Google Scholar databases. All papers published before June 2020 were included in the review. A large set of keywords was used including growth factor, tyrosine kinase receptor, diabetic PAD, angiogenesis, blood flow reperfusion, endothelial dysfunction, endothelial cells, perivascular cells, hindlimb ischemia, phosphatase, and hyperglycemia.

## Introduction

Current data reveals that more than 400 million people are suffering from diabetes mellitus (DM) and projections estimate that numbers of this world-wide issue will almost triple by 2030 (1). DM is a major risk factor of vascular complications altering both micro- and macrovascular beds. Peripheral artery disease (PAD) belongs to the macrovascular complications of diabetes with an estimated prevalence of 20% in diabetic patients over 40 years of age reaching 29% in patients over 50 years of age (2). The severity and the duration of DM are important predictors of both the incidence and the extent of PAD which appears more frequently, more severe, and at a younger age compared to non-diabetic PAD (3). Diabetic patients with PAD are also less responsive to revascularization procedures leading to a dramatic 5 to 15 times higher risk of major amputation (4). The pathophysiology of PAD is characterized by obstructive atherosclerosis of the lower limb arteries gradually reducing blood flow to the tissues and ultimately leading to critical ischemia (5). Despite both major angiogenic inducers, hypoxia and oxidative stress, being elevated in diabetic PAD, collateral vessel development is insufficient to support the loss of blood flow through occluded arteries. The deregulation of growth factors represents a hallmark of diabetic vascular complications occurring differentially in various tissues (6). Although excessive activity and overproduction of pro-angiogenic growth factors contribute to the progression of diabetic nephropathy or retinopathy, the angiogenic paradox suggests that diabetic PAD and myocardial infarction (MI) are rather characterized by defects in the production and/or responsiveness to pro-angiogenic growth factors (7). Thus, identifying the underlying molecular mechanisms that are involved in the deregulation of pro-angiogenic factors has generated increased interest in the context of diabetic PAD. Protein tyrosine phosphatases (PTP) are critical regulators of pro-angiogenic tyrosine kinase receptor (RTK) signaling (8). Not only do PTP shut down RTK, they also finely modulate tyrosine phosphorylation of these receptors to specifically regulate several cellular angiogenic properties. Thus, any slight dysregulation in PTP expression or activity will trigger pathological angiogenesis. Evidence has emerged concerning the role of PTP in the development of diabetes and the progression of diabetic vascular complications including nephropathy (9) and retinopathy (10). Therefore, this review aims to summarize and discuss the deregulation of the main growth factors in diabetic PAD with a translational view from clinical studies to mechanistic preclinical models. We next sought to discuss the role of PTP in the regulation of angiogenic pathways and highlight how PTP expression and activity are regulated in the hypoxic environment of diabetic PAD.

## Deregulation of Pro-Angiogenic Factors in Diabetic PAD

**Figure 1:**
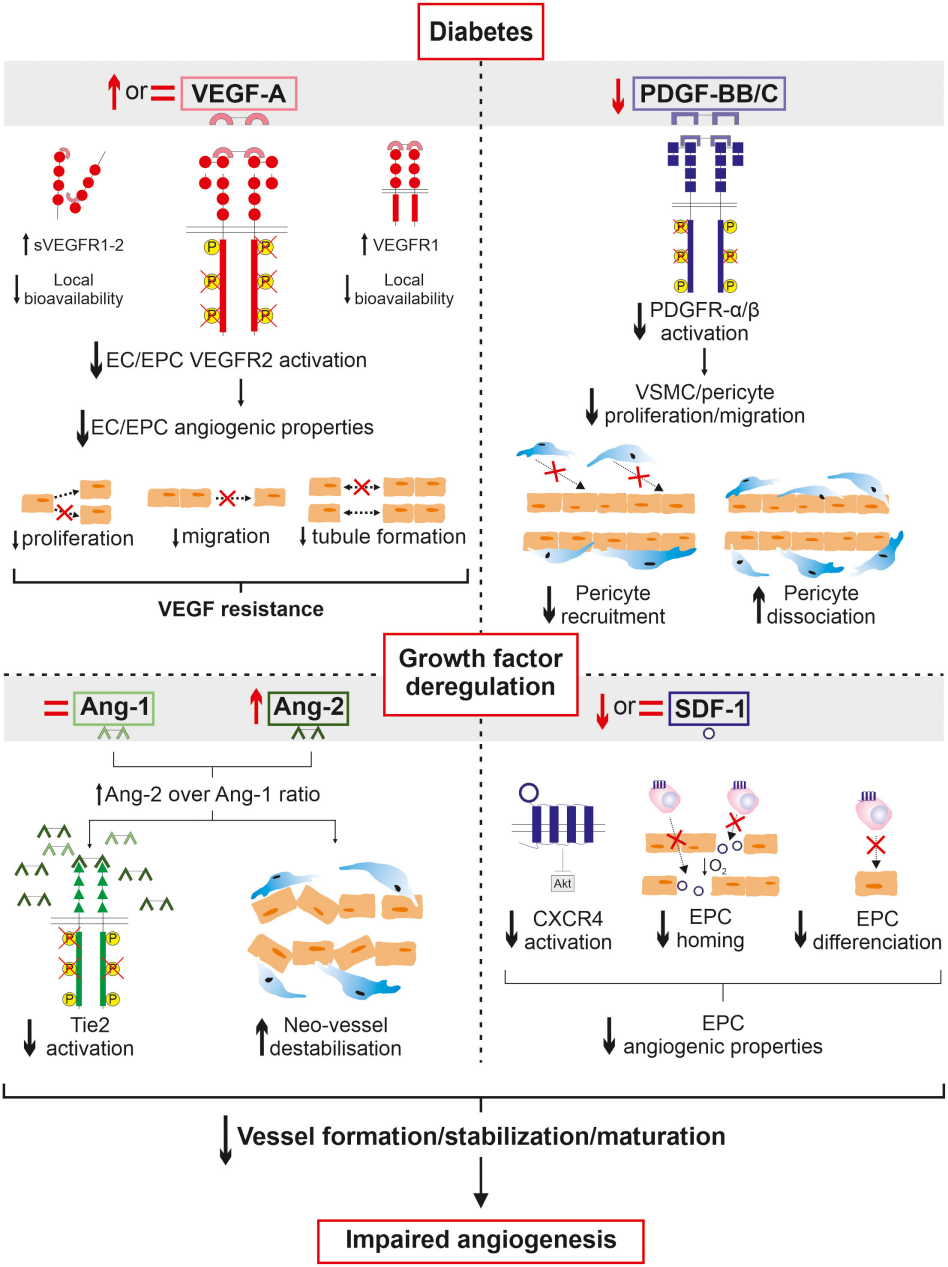
Overview of growth factor deregulation in diabetic PAD. A history of diabetes deeply altered circulating growth factor expression in response to ischemia in PAD. Despite an increased production of VEGF, decreased cell responses to VEGF are observed in diabetes. The VEGF resistance paradox could be partially attributed to a lack of local bioavailability due to increased expression of both the soluble forms of VEGF receptor and VEGFR1 acting together as decoys to sequestrate VEGF. In addition, VEGF signaling is impaired leading to the alteration of endothelial cell angiogenic properties which fail to initiate the angiogenic process upon VEGF stimulation. Concurrently, PDGF production and PDGFR signaling are decreased therefore contributing to reduced pericyte/vascular smooth muscle cell coverage of neo-vessels in addition to promoting their dissociation. Angiopoietin balance is also deregulated to promote Ang-2 over Ang-1 expression. This implies decreased activation of Tie2 receptor subsequently leading to Ang-2-induced destabilization and regression of neo-vessels. Finally, SDF-1 production and CXCR4 activation are altered resulting in endothelial progenitor cell dysfunctions and consequently failing to efficiently migrate to the ischemic environment and trigger endothelial differentiation. Taken together, the deregulation of growth factors in diabetes negatively affects all fundamental steps of neo-vessel generation to ultimately alter angiogenesis in the ischemic lower limb of patients suffering from PAD.

Angiogenesis is a complex, dynamic, and well-coordinated physiological process leading to the formation of new vessels from pre-existing vasculature in response to ischemia. This process involves temporal interactions between multiple growth factors and vascular cells, which have been extensively summarized in excellent reviews (11, 12). Briefly, vascular endothelial growth factor (VEGF), platelet-derived growth factor (PDGF), angiopoietins (Ang-1 and Ang-2), and stromal cell-derived factor 1 (SDF-1) are the main actors in angiogenesis and the most studied in diabetic PAD (Table 1). Overall, SDF-1 is required for the recruitment of undifferentiated endothelial progenitor cells (EPC) to the hypoxic environment, while VEGF initializes the proliferation and sprouting of endothelial cells (EC) to form immature neo-vessels. In a final step, PDGF and Ang-1 drive the recruitment of pericytes and vascular smooth muscle cells (VSMC) which wrap around and stabilize neo-vessels to develop mature capillaries. The next section provides an overview of the current understanding of main growth factor deregulation in diabetic PAD (Figure 1). Clinical studies will be briefly summarized while preclinical models (*in vitro* and *in vivo*) will be reported to highlight the underlying molecular mechanisms leading to the alteration of the angiogenic process following hindlimb ischemia in diabetes. Findings based on other diabetic macrovascular complications sharing a similar pathophysiology as PAD will be reported where preclinical data was not available in the context of diabetic PAD.

**Table 1:** Clinical investigations of growth factors in diabetic PAD.

**References**	**Experimental design**	**Endpoints**	**Main findings**
Waltenberger et al. (13)	Healthy (*n* = 14) T1D (*n* = 10) T2D (*n* = 6)	VEGF	^*^↑ Diabetic vs. healthy = T1D vs. T2D
Hochberg et al. (14)	Healthy (*n* = 18) Healthy CLI (*n* = 8) Diabetic (*n* = 10) Diabetic CLI (*n* = 8)	VEGF mRNA mono. VEGF hypoxic induction	= all groups ^*^↑ Diabetic-CLI vs. diabetic
Blann et al. (15)	Healthy (*n* = 70) PAD (*n* = 70) Healthy (n=14) T2D w/o CVD (*n* = 14) T2D w/ CVD (*n* = 14)	VEGF, sVEGFR1	^*^↑ VEGF PAD vs. healthy ^*^↑ VEGF T2D w/ vs. T2D w/o CVD ^*^↓ sVEGFR1 PAD vs. healthy = sVEGFR1 T2D w/ and T2D w/o CVD
Makin et al. (16)	Healthy (*n* = 50) PAD (*n* = 234) [w/ diabetes (*n* = 81)]	VEGF, sFlt1	^*^↑ VEGF PAD vs. healthy = VEGF PAD vs. PAD w/diabetes
Mahdy et al. (17)	Heathy (*n* = 15) T2D (*n* = 10) T2D w/ macrovascular comp.	VEGF	^*^↑ T2D vs. healthy ^*^↑ T2D w/ macrovascular comp. vs. T2D
Orrico et al. (18)	Healthy (*n* = 29) CLI (*n* = 33) [w/ T2D (*n* = 22)]	VEGF serum and mRNA arterial wall	^*^↑ VEGF serum and mRNA CLI vs. healthy = VEGF serum and mRNA CLI vs. CLI w/diabetes
Bleda et al. (19)	T2D IC (*n* = 32) T2D CLI (*n* = 38)	VEGF	^*^↑ T2D CLI vs. T2D IC
Zakareia (20)	Healthy (*n* = 30) T2D (*n* = 30) T2D w/ PAD (*n* = 30)	VEGF	^*^↑ T2D w/ PAD vs. T2D and healthy ^*^positive correlation VEGF/ABI in T2D w/ PAD
Wieczór et al. (21)	Healthy (*n* = 30) PAD w/ T2D (*n* = 15) PAD w/o T2D (*n* = 31)	VEGF sVEGFR1 sVEGFR2	^*^↑ VEGF PAD w or w/o T2D vs. healthy ^*^↓ sVEGFR1 PAD w or w/o T2D vs. healthy = all factors PAD w or w/o T2D
Yeboah et al. (22)	T2D w/ prior CVD events (n=36) T2D w/o CVD events (*n* = 41)	VEGF, FGF-2, HGF, PDGF-BB	^*^↓ FGF-2 and PDGF-BB T2D w/ prior CVD events vs. T2D w/o CVD events
Lim et al. (23)	Healthy (*n* = 34) T2D w/o CVD (*n* = 65) T2D w/ CVD (*n* = 38)	VEGF, Ang-1, Ang-2	^*^↑ VEGF and Ang-2 T2D w/ or w/o CVD vs. healthy ^*^positive correlation VEGF and Ang-2 with HbA1c in T2D w/o CVD
Lim et al. (24)	Healthy (n=35) T2D w/o CVD (*n* = 56) T2D w/ CVD (*n* = 41)	VEGF, Ang-1, Ang-2	^*^↑ VEGF and Ang-2 T2D w/ or w/o CVD vs. healthy = all factors T2D w/ or w/o CVD
Findley et al. (25)	Healthy (n=23) IC (*n* = 23) [w/ diabetes (*n* = 4)] CLI (*n* = 23) [w/ diabetes (*n* = 9)]	VEGF, PIGF, Ang-2, sTie2sVEGFR1,	^*^↑ VEGF, Ang-2, sTie2 PAD vs. healthy ^*^↑ VEGF, sTie2 CLI vs. IC independently of diabetes
Li et al. (26)	Healthy (*n* = 50) T2D (*n* = 120) [w/ macrovascular comp. (*n* = 32)] [w/ microvascular comp. (*n* = 52)] [w/o macrovascular comp. (*n* = 36)]	Ang-1, Ang-2	^*^↑ Ang-2 T2D vs. healthy ^*^↑ Ang-2 T2D w/ macrovascular comp. vs. w/o macrovascular comp. = Ang-1 all groups
Siddiqui et al. (27)	T2D (*n* = 80) [w/ CVD (*n* = 29.1%)]	Ang-2, sTie2	^*^↑ Ang-2 in T2D w/ CVD ^*^positive correlation sTie2 with HbA1c
Rasul et al. (28)	T2D (*n* = 329) [w/ CVD (*n* = 62)]	Ang-2	^*^↑ Ang-2 in T2D w/ CVD

ABI, ankle brachial index; Ang-1, angiopoietin-1; Ang-2, angiopoietin-2; CLI, critical limb ischemia; CVD, cardiovascular disease; comp, complications; EGF, epidermal growth factor; FGF-2, fibroblast growth factor 2; HbA1c, glycated hemoglobin; HGF, hepatocyte growth factor; IC, intermittent claudication; PAD, peripheral artery disease; PDGF-BB, platelet-derived growth factor; PIGF, Phosphatidylinositol-glycan biosynthesis class F protein; sTie2, soluble Tie2 receptor; SDF-1, stromal cell-derived factor 1; sVEGFR1, soluble vascular endothelial growth factor receptor 1; sVEGFR1, soluble vascular endothelial growth factor receptor 2; T2D, type II diabetic; VEGF, vascular endothelial growth factor; w/, with; w/o, without. ^*^↑, significant increase; ^*^↓, significant decrease; =, no changes. Growth factor were evaluated in serum otherwise stated.

### Clinical Investigations

#### VEGF

From the extensive list of pro-angiogenic factors, VEGF is by far the most clinically investigated. As a result of blunted cellular response to hypoxia, hypoxia-inducible factor-1 alpha (HIF-1α) itself and most of its target genes, including VEGF, are reduced in a diabetic state (29). Thus, a slightly lower non-significant expression of VEGF-A was observed in the serum of type 2 diabetic (T2D) PAD patients compared with non-diabetic PAD individuals (21). On the other hand, PAD is characterized by a tremendously favorable ischemic environment triggering VEGF production and an ample number of investigations have suggested that patients with PAD and coexisting diabetes display higher or at least unchanged serum levels of VEGF-A (Table 1) (13–20, 22–25). Therefore, the altered collateral vessel formation in diabetic PAD might be attributed to defects in downstream signal transduction rather than in VEGF production in response to ischemia (13, 14). This hypothesis is further supported by increased levels of both soluble vascular endothelial growth factor receptor 1 (sVEGFR1) and 2 (sVEGFR2) in the serum of T2D patients with PAD compared with nondiabetic PAD individuals (21) that could act as decoys to sequestrate VEGF-A (15). Collectively, the lack of significant improvement of the PAD following exogenous administration of VEGF could be attributed to decreased local bioavailability and unresponsiveness of VEGF in diabetic individuals.

#### PDGF

Unlike VEGF, the impact of hyperglycemia on PDGF expression and activation in the context of PAD has been poorly clinically investigated. To date, only one pilot study has reported lower serum levels of PDGF-BB in T2D patients with a previous cardiovascular event compared to those that have not (22) (Table 1). Although this investigation did not provide definitive evidence of additive effects of diabetes, it suggested that PDGF-BB could be a predictive marker of cardiovascular diseases (CVD) in T2D.

#### Angiopoietins

Two consecutive cross-sectional studies investigated the serum levels of Ang-1 and Ang-2 in control and T2D patients, with or without CVD, including PAD (23, 24) (Table 1). Only Ang-2 serum levels were significantly raised in T2D patients compared to controls, which correlated with an increased index of endothelial dysfunction (24). Interestingly, Ang-2, but not Ang-1, serum levels were increased in T2D patients compared to healthy controls and positively correlated with circulating glycated hemoglobin (HbA1c) (23). In addition, several clinical investigations showed that Ang-2 levels were increased in T2D subjects with macrovascular complications compared to those without (26–28). Increased Ang-2 levels were also reported in the serum of PAD patients along with the soluble Tie2 (sTie2) receptor. Interestingly, sTie2 levels were correlated with an increased severity of PAD but were not associated with several cardiovascular risk factors including diabetes (26). Similarly, Ang-2 but not sTie2 levels were significantly associated with the presence of macrovascular complications including PAD (28). Adding to hyperglycemia-induced increase Ang-2 serum levels, these two last studies raised the hypothesis that sTie2 could be a marker of PAD severity regardless of diabetes.

#### SDF-1

The role of SDF-1 in diabetic vascular complications has gained interest since an early study found a significant decrease in the number of circulating EPC in T1D patients compared to controls (30). The reduction in circulating EPC was further diminished in diabetic subjects with PAD compared to non-diabetic individuals with PAD, demonstrating a noxious additive effect of hyperglycemia (31). Interestingly, Egan *et al*. reported lower numbers of CXCR4-positive circulating cells, SDF-1's main receptor in T2D patients which was further decreased in T2D individuals suffering from peripheral atherosclerosis (32).

### *In vivo* Studies

Animal studies are based on models of hindlimb ischemia (HLI) that mimic the pathophysiology of PAD and allow to assess ischemia-induced angiogenesis/arteriogenesis as well as subsequent blood flow reperfusion. Several different surgical approaches are used to induce ischemia in the lower limb of small animals (33, 34). The easier approach is a one-point distal ligation of the femoral artery while the most reported is a distal and proximal ligation followed by the excision between these two ligation points. These methods will result in a mild (ligation) and acute (ligation and excision) ischemia that ultimately influence both the temporality and the efficiency of blood flow reperfusion in the ischemic limb (33, 34). Although these variations might be responsible on the variability observed in local therapeutic studies, no conflicting results were found in the angiogenic outcomes reported in the context of this review.

#### VEGF

Early studies reported decreased or unchanged expression of VEGF in the ischemic muscle of non-obese T1D and *db*/*db* T2D mice, which was illustrated by a significant reduction in capillary density and blood flow reperfusion 35 days post HLI-surgery (35, 36). In the same time frame, blood flow reperfusion was also dampened in high-fat diet T2D mice despite ischemia-induced upregulation of VEGF production (37). Interestingly, sVEGFR1 and VEGFR1 expression were gradually increased and correlated with significantly higher VEGF binding in the diabetic ischemic muscle compared to that of non-diabetic mice sustaining the hypothesis of a locally limited VEGF bioavailability suggested by clinical studies (13, 14). In addition to an alteration in VEGF production, a lower expression and/or activation of its receptor VEGFR2 were reported in the 28-day ischemic muscle of *db*/*db* T2D mice (38) as well as in streptozotocin (STZ)-induced T1D mice 3, 14 and 28 days post HLI-surgery, respectively (39–42). This was illustrated by reduced capillary density and blood reperfusion suggesting angiogenic defects in the diabetic lower limb. The reduced phosphorylation of VEGFR2 was notably dependent on the activation of protein kinase C delta (PKCδ) and angiotensin II receptor type 2 (ATR2) in a diabetic state (41, 42). In line with the impaired activation of VEGFR2, a decreased phosphorylation of several downstream effectors of the VEGF pathway including protein kinase B (Akt) (30, 34–36), endothelial nitric oxide synthase (eNOS) (40, 43, 44) and extracellular signal-regulated kinase (ERK) (41) was reported in the ischemic muscle of both T1D and T2D mice.

#### PDGF

In the HLI mouse model, a decreased expression of PDGF-BB was reported in the ischemic muscle of both STZ-induced T1D and *ob*/*ob* T2D mice compared to wild-type counterparts 7 days post-surgery which was correlated with an increased activation of PKC-α (45, 46). The impaired blood flow reperfusion in the ischemic diabetic limb was associated with an apparent dissociation of pericytes from the endothelial capillary tube (45). Interestingly, a reduction of PDGF expression along with an alteration of PDGF receptor beta (PDGFR-β) phosphorylation was found in the ischemic muscle of STZ-induced T1D mice compared to controls 28 days post-ligation of the femoral artery reflecting persisting defects in PDGF signaling (40).

#### Angiopoietins

Unfortunately, no HLI-based animal studies have yet been conducted to elucidate the role of angiopoietins in the revascularization of diabetic lower limbs. Although, interesting findings were observed in MI, a well-known diabetic macrovascular complication in the same category as PAD. Hence, the expression of Ang-2 was significantly upregulated in the ischemic heart of both T2D and T1D mice compared to ischemic controls while Tie2 expression and signaling were downregulated and Ang-1 was unaffected (47, 48). Hyperglycemia-induced disruption of Tie2 signaling by favoring Ang-2 expression over Ang-1 promoted EC apoptosis that subsequently aggravated the size of myocardial infarction (47). Importantly, Ang-1-induced recruitment of VSMC and EC coverage were impaired in the diabetic ischemic heart suggesting alterations in the maturation process of capillaries. This was illustrated by a significant reduction of myocardial capillary density, arteriole formation and vessel outgrowth (48).

#### SDF-1

In a HLI model, the recruitment of EPC was completely abolished in T2D rats due to a decrease in SDF-1 serum concentrations 3 days post-surgery that was illustrated by an impaired blood flow recovery in the diabetic limb (31). Interestingly, bone marrow progenitor cells from T1D mice were unable to differentiate into an endothelial phenotype following SDF-1 stimulation thus showing that hyperglycemia also deregulates the CXCR4 signaling pathway (49). An elegant study recently found a significant reduction of SDF-1 levels in both serum and ischemic muscle of STZ-induced diabetic mice 3 days post femoral artery ligation. This was accompanied with blunted activation of Akt leading to decreased recruitment of EPC to the ischemic sites triggering insufficient blood reperfusion (50).

### Cell-Based Mechanistical Studies

#### Endothelial cells/Endothelial Progenitor Cells

At the cellular level, a recent study showed increased production of VEGF-A_165_ but decreased mRNA expression and blunted phosphorylation of extracellular signal-regulated kinases (ERK) 1/2 in HUVEC simultaneously exposed to hyperglycemia and CoCl2-induced hypoxia compared to controls leading to reduced migration (51). This could be attributed to a downregulation of VEGFR2 expression under concurrent hypoxic and hyperglycemic environments (39). Recently, exposure of HUVEC to methylglyoxal (MGO), a glucose metabolite, reduced the expression and production of PDGF-BB while raising VEGF-A expression, which ultimately resulted in pericyte recruitment inhibition (52). In time course experiments on EC exposed to high glucose, both Akt and eNOS activation were decreased and delayed overtime as the expression of Ang-2 increased compared to normal glucose concentrations (48). Despite unaltered expression of Ang-1, Ang-1-stimulated Tie2 activation was decreased in HUVEC exposed to high glucose levels leading to reduced Akt phosphorylation (53). However, the angiogenic outcomes related to diabetes-induced angiopoietin imbalance remain to be identified.

Several alterations in growth factor expression have also been reported in the EPC niche in hyperglycemia. Exposure of EPC to high glucose blunted the activation of Akt and eNOS, which contributed to the inhibition of VEGF-induced EPC proliferation, migration, or tubule formation and ultimately triggered abnormal neovasculogenesis (54, 55). Interestingly, mRNA levels of Ang-1 were decreased in the EPC from healthy volunteers. Furthermore, exposing these cells to hyperglycemia and exogenous Ang-1 improved EPC angiogenic properties (56). Similarly, EPC extracted from diabetic mice exhibited low expression of Ang-1 compared to non-diabetic counterparts (57). Finally, EPC exposed both to hypoxia and high glucose levels exhibited decreased protein levels of SDF-1 and Akt phosphorylation, which was associated with an impairment of SDF-1-induced tubule formation (50).

#### Vascular Smooth Muscle Cells/Pericytes

In contrast to the endothelial lineage, only few studies have investigated the molecular mechanisms underlying growth factor deregulation in hyperglycemic perivascular cells. The expression of PDGFR-α and β were decreased in VSMC exposed to high glucose levels due to the upregulation of PKC-α. On the other hand, PDGF-C was only decreased in VSMC suggesting that perivascular cells might be more responsible for decreased expression of PDGF-C previously reported in the whole diabetic muscle (58, 59). Similarly, non-toxic MGO concentrations altered PDGF-BB-induced PDGFR-β and ERK1/2-activation as well as the subsequent proliferation of VSMC (60).

#### Monocytes

Monocytes extracted from diabetic patients with CLI do not exhibit impairment in the ability to produce VEGF in hypoxic conditions (14). Nevertheless, monocytes extracted from diabetic subjects presented severe impairment in the chemotaxis response to VEGF stimulation despite a significantly higher concentration of VEGF in the serum of diabetic patients (13). However, functional angiogenic outcomes related to monocyte dysfunction in diabetes still needs to be evaluated.

## Emerging Role of Phosphatases in the Regulation of Angiogenesis

**Figure 2:**
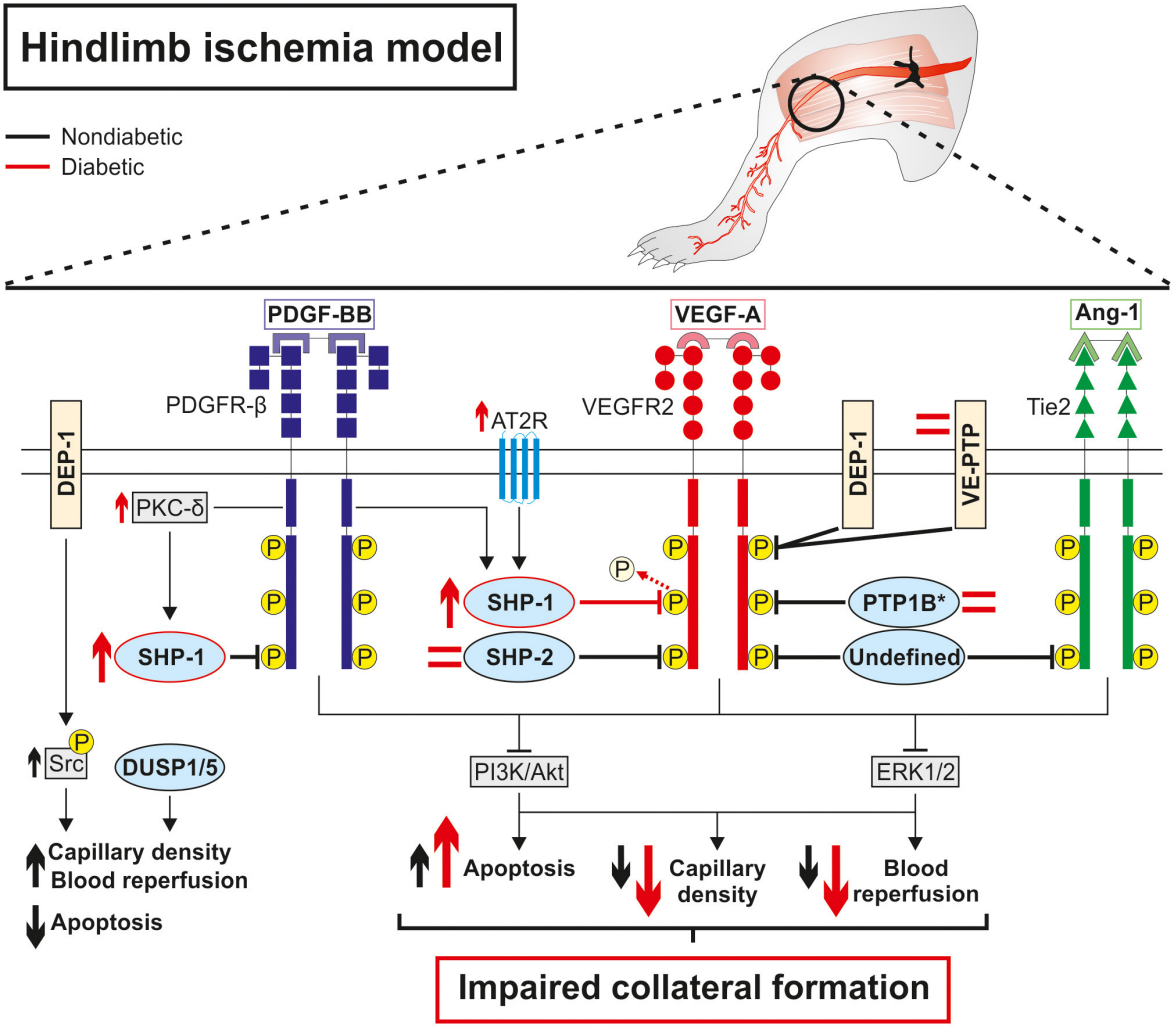
Diabetes impaired collateral vessel formation by deregulating phosphatase expression and activity in a hindlimb ischemia model. Overall, protein tyrosine phosphatases are direct negative regulators of pro-angiogenic receptors tyrosine kinase PDGFR-β, VEGFR2, and Tie2 at baseline, which limits collateral vessel formation in the hindlimb ischemia model. In contrast, DEP-1 exerted indirect positive regulation of VEGFR2 by dephosphorylating inhibitory Y529 on Src while DUSP1/5 promoted capillary formation and blood reperfusion through the reduction of endothelial cell apoptosis in an RTK-independent way. Diabetes activates the PKC-δ isoform and the angiotensin II receptor type 2 that in turn increases the expression of SHP-1 and its phosphatase activity against VEGFR2, ultimately leading to blunted downstream signaling and aggravating impaired collateral vessel formation following ischemia. Conversely, SHP-2, PTP1B, and VE-TPT expressions are unchanged in endothelial cells (*PTP1B) or whole diabetic ischemic muscle compared to non-diabetic counterparts suggesting a lesser role of these phosphatases in diabetic PAD. The regulation and the subsequent influence of DEP-1 and DUSP1/5 in hindlimb angiogenesis still needs to be investigated in the context of diabetic PAD.

**Figure 3:**
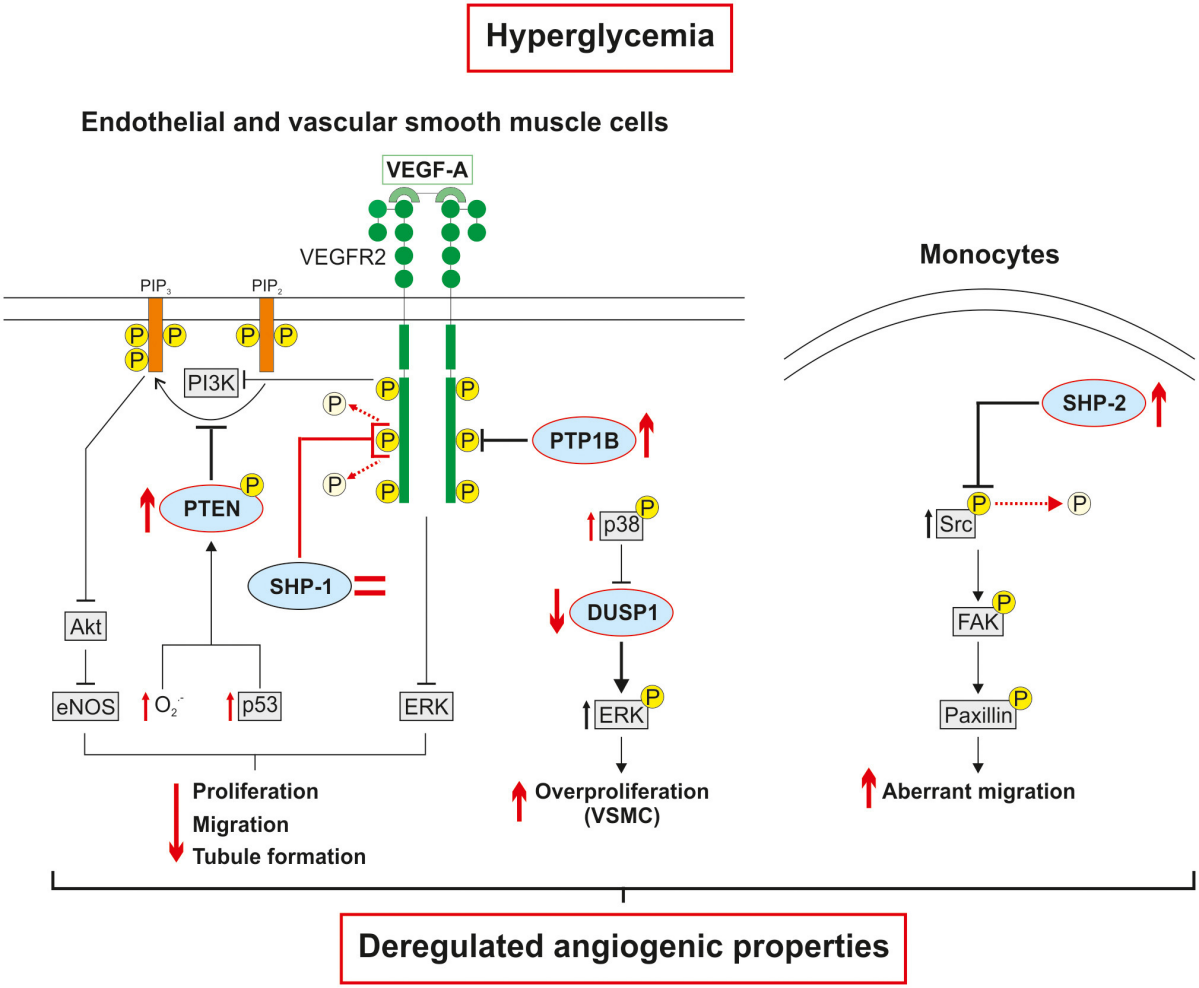
Hyperglycemia-induced phosphatase deregulation is associated with dysfunctions in cellular angiogenic responses. The expression of PTP1B, a negative regulator of VEGFR2, is increased in hyperglycemia. Conversely, the expression of SHP-1 is unchanged in endothelial cells exposed to high glucose. However, SHP-1 phosphatase activity and interaction with VEGFR2 are increased leading to decreased VEGFR2 activation and downstream signaling. The hyperglycemia-induced overproduction of free radicals and upregulation of p53 contribute to increased PTEN expression and phosphorylation which in turn reduced the production of phosphatidylinositol-3,4,5-trisphosphate (PIP_3_). The direct deactivation of VEGFR2 signaling followed by the indirect decrease in the production of downstream effectors by phosphatases, ultimately impair the pro-angiogenic properties of endothelial cells. In vascular smooth muscle cells, high glucose levels increase p38 expression and activation, which reduces DUSP1 expression, leads to sustained overactivation of ERK and subsequent abnormal proliferation. In monocytes, hyperglycemia increases the expression of SHP-2 and its phosphatase activity on an inhibitory tyrosine residue of Src kinase. The overactivation of Src activates the FAK/paxillin axis resulting in aberrant and ineffective migration of monocytes subjected to VEGF stimulation.

As described above, the expression and activation of growth factors are deeply impaired in diabetes leading to blunted pro-angiogenic signaling pathways and poor collateral vessel formation in response to a PAD ischemic environment. Pro-angiogenic RTK, including VEGFR, PDGFR and Tie2, are mainly regulated by a fine balance between tyrosine phosphorylation and dephosphorylation. Any slight alteration in the equilibrium between protein tyrosine kinase and protein tyrosine phosphatase (PTP) activity will trigger abnormalities in cellular processes including proliferation, migration, and differentiation, thereby resulting in a global deregulation of angiogenesis (Table 2). Additionally, phosphatases can directly act on several downstream RTK effectors to modulate specific pathways and cell functions (87). Over the years, the influence of PTP in both micro and macrovascular complications of diabetes has been progressively discovered. Hyperglycemia-induced modification of PTP expression or activity has been shown to deregulate the insulin (88), nephrin (89), and PDGFR-β (10) pathways contributing to insulin resistance, nephropathy, and retinopathy, respectively. Early evidence concerning the potential role of PTP in the regulation of collateral vessel angiogenesis emerged when administration of broad-spectrum PTP inhibitors improved blood flow reperfusion following HLI in mice (Figure 2) (90, 91). Given that several PTP have been identified in the vascular lineage since (92), the following section summarizes and discusses evidence of PTP contribution in the regulation of pro-angiogenic RTK as well as their emerging role in the pathophysiology of PAD (Table 3). In addition, a focus was made to highlight current knowledge concerning the effects of hyperglycemia in the modulation of PTP and related angiogenic outcomes at the mechanistical cellular level (Figure 3) and in the context of HLI models reflecting the microenvironment of PAD (Table 4).

**Table 2:** Baseline regulation of RTK by phosphatases in angiogenesis.

		**RTK regulation**				
**Phosphatase**	**Cell type**	**VEGFR2**	**PDGFR-β**	**Tie2**	**Angiogenic effects**	**References**
PTP1B	EC	Negative	-	-	↓ Proliferation ↓ Migration ↓ Tube formation	(61, 62)
			-	-	↓ Proliferation	(63)
SHP-1	EC	Negative	-	-	↓ Proliferation	(64–67)
		Negative	-	-	↓ Tube formation ↑ Apoptosis	(68)
SHP-2	EC	Negative	-	-	↓ Migration	(69, 70)
VE-PTP	EC	Negative Positive	-	-	↓ Proliferation ↑ Tube formation	(71)
		Negative	-	-	↓ Proliferation	(72)
		-		Negative	↓ Proliferation	(73)
DEP-1	Fib/EC	-	Negative	-	↓ Migration	(74)
	EC	Negative	-	-	↓ Proliferation	(75)
					↓ Survival	(76)
					↓ Proliferation ↓ Tube formation	(77)
TC-PTP	Fib	-	Negative	-	↓ Migration	(78)
	EC	Negative	-	-	↓ Proliferation ↓ Migration ↓ Sprouting	(79, 80)
PTEN	EC	Negative indirect (↓ PI3K/Akt)	-	-	↓ Proliferation ↓ Migration ↓ Tube formation	(81)
	VSMC	-	Negative indirect (↓ PI3K/Akt)	-	↓ Proliferation ↓ Migration	(82)
DUSP1	EC	Positive indirect (MAPKs)	-	-	↑ Migration ↑ Sprouting	(83)
		Positive indirect (MAPKs)	-	-	↑ Proliferation	(84)
		Positive indirect (MAPKs)	-	-	↑ Tube formation ↑ Migration	(85)
DUSP4	Fib	-	Positive indirect (MAPKs)	-	↑ Proliferation ↓ Apoptosis	(86)
DUSP5	EC	Negative indirect (MAPKs)	-	-	↓ Proliferation	(84)

*Akt, protein kinase B; EC, endothelial cell; Fib, fibroblast; MAPK, mitogen-activated protein kinase; PDGFR-β, platelet-derived growth factor receptor beta; PI3K, phosphoinositide 3-kinase; RTK, receptor tyrosine kinase; Tie2, tyrosine kinase with immunoglobulin-like and EGF-like domains 2; VEGFR2, vascular endothelial growth factor receptor 2; VSMC, vascular smooth muscle cell. -, not evaluated; ↓, decrease; ↑, increase*.

**Table 3:** Baseline regulation of collateral angiogenesis by PTP in HLI models.

**Phosphatase**	**HLI induction**	**Inhibition**	**RTK**	**Angiogenic effects**	**Overall reg**.	**References**
Untargeted	Excision of the femoral artery	Non-selective inhibitor	Tie2	↑ Tie2 activation ↑ Reperfusion (2–4 weeks)	Negative	(91)
Untargeted	Distal and proximal ligation of the femoral artery	Non-selective inhibitor	VEGFR2	↑ VEGFR2 signaling ↑ Capillary density (3 weeks)	Negative	(92)
PTP1B	Ligation and excision of the femoral artery	EC-specific KO	VEGFR2	↑ Reperfusion (2 weeks)	Negative	(93)
SHP-1	Excision of the femoral artery	I.M siRNA	VEGFR2	↑ Capillary density (3 weeks)	Negative	(68)
DEP-1	Distal and proximal ligation of the femoral artery	Whole-body KO	Undefined	None (1 week)	-	(94)
	Excision of femoral and saphenous arteries	Whole-body KO	VEGFR2	↓ Reperfusion ↓ Capillary density ↑ Ischemic damage (2 weeks)	Positive	(95)
DUSP1	Excision of the femoral artery and vein	Whole-body KO	Undefined	↓ Reperfusion (days 3–7) ↓ Vascular complexity (day 7)	Positive	(96)
DUSP5	Ligation and excision of the femoral artery	R.O siRNA	Undefined	↓ Reperfusion ↑ Limb necrosis (2 weeks)	Positive	(84)

*EC, endothelial cells; I.M, intramuscular; KO, knockout; reg., regulation; R.O, retro-orbital; RTK, receptor tyrosine kinase; Tie2, tyrosine kinase with immunoglobulin-like and EGF-like domains 2; VEGFR2, vascular endothelial growth factor receptor 2; ↓, decrease; ↑, increase*.

**Table 4:** Hyperglycaemia-induced PTP modulation.

**Phosphatase**	**Cell type**	**HLI induction**	**RTK**	**HG effects**	**Angiogenic outcomes**	**References**
PTP1B	Whole muscle	n/a	Undefined	*In vivo* ↑ Expression ↑ Activity	-	(97)
	EC	n/a	Untargeted	*In vitro* ↑ Expression	-	(62)
	Ischemic muscle (28 days)	Distal and proximal ligation of the femoral artery	VEGFR2 PDGFR-β	*In vivo* = Expression	-	(41)
SHP-1	Ischemic muscle (28 days)	Distal and proximal ligation of the femoral artery	VEGFR2 PDGFR-β	*In vivo* ↑ Expression	↓ Reperfusion ↓ Capillary density	(41)
	EC muscle Ischemic muscle (28 days)	n/a Distal and proximal ligation of the femoral artery	VEGFR2	*In vitro* = Expression ↑ Binding ↑ Activity *In vivo* ↑ Expression ↑ Binding	↓ Proliferation ↓ Migration ↓ Reperfusion ↓ Capillary density	(42)
SHP-2	Ischemic muscle (28 days)	Distal and proximal ligation of the femoral artery	VEGFR2 PDGFR	*In vivo* = Expression	-	(41)
	Ischemic muscle (28 days)	Distal and proximal ligation of the femoral artery	VEGFR2	*In vivo* = Expression	-	(42)
	Monocytes	n/a	VEGFR1	*In vitro* ↑ Expression ↑ Activity	↓ Migration ↓ Chemotaxis	(98)
VE-PTP	Ischemic muscle (28 days)	Distal and proximal ligation of the femoral artery	VEGFR2	*In vivo* = Expression	-	(42)
PTEN	EC	n/a	Untargeted	*In vitro* ↑ Expression	-	(82) (99)
DUSP1	VSMC	n/a	Untargeted	*In vitro* ↓ Expression	↑ Proliferation	(100)

*RTK, receptor tyrosine kinase; PDGFR-β, platelet-derived growth factor receptor beta; HG, hyperglycemia; EC, endothelial cells; VSMC, vascular smooth muscle cells; VEGFR1-2, vascular endothelial growth factor receptor 1-2; n/a, not applicable; -, not evaluated; ↓, decrease; ↑, increase; =, unchange*.

### Protein Tyrosine Phosphatase 1B

Protein tyrosine phosphatase 1B (PTP1B, gene: *PTPN1*) is an intracellular ubiquitous PTP. The systemic deletion of PTP1B results in viable and fertile mice with enhanced insulin sensitivity and accelerated wound healing (88). PTP1B has been initially described as a critical negative regulator of insulin signaling through its interaction with the insulin receptor (101). Early evidence in the role PTP1B in angiogenesis has emerged when a PTP1B inhibitor was shown to enhance VEGF-mediated VEGFR2 activation, migration, and proliferation of EC (61). The overexpression of PTP1B in EC inhibited VEGF-induced phosphorylation of VEGFR2 and signaling, which lead to altered EC proliferation whereas EC extracted from *Ptpn1*^−/−^ mice exhibited increased angiogenic properties (63). Interestingly, hyperglycemia was found to increase PTP1B expression in macrovascular EC leading to endothelial dysfunction (62).

In the setting of PAD, PTP1B expression and activity were upregulated by hypoxia in the ischemic limb of mice 7 days post-HLI surgery (63) and endothelial-specific PTP1B deletion was sufficient to significantly improve blood flow reperfusion and capillary density (93). These findings emphasized PTP1B as a negative regulator of ischemia-induced angiogenesis through inhibition of the VEGF pathway in the endothelial lineage. Unfortunately, the regulation and influence of PTP1B remain to be clarified in a diabetic PAD environment. Indeed, PTP1B expression and activity was upregulated in skeletal muscle of Goto-Kakizaki T2D rats (97) while its expression remained unchanged in the ischemic muscle of STZ-induced T1D mice 28 days post-ligation of the femoral artery (41). These data suggest that the type of diabetes, presence and duration of hypoxia could influence PTP1B regulation in the lower limb.

However, PTP1B levels and activity were increased in the wounded skin of STZ-induced T1D (102) and *ob*/*ob* T2D (103) mice compared to wild-type counterparts, which was associated with impaired neovascularization and subsequent delayed wound healing, a well-known complication of diabetic PAD (104). Administration of a selective inhibitor significantly accelerated the wound healing process by decreasing PTP1B activity, which promoted a local recovery of VEGFR2 phosphorylation that contributed to the restoration of EC angiogenic properties (102, 103). Hence, diabetic wound-healing studies highlighted that hyperglycemia exacerbated PTP1B-induced downregulation of angiogenesis in macrovascular complications.

### Src Homology Region 2 Domain-Containing Phosphatase-1

Src homology region 2 domain-containing phosphatase-1 (SHP-1, gene: *PTPN6*) is a cytoplasmic tyrosine phosphatase. SHP-1 null mice showed chronic systemic inflammation and died within 3 weeks of birth from interstitial pneumonia (105). Initially, SHP-1 was found to have a role in VEGF signaling due to its association with VEGFR2 following VEGF-A stimulation in EC (106). Further studies confirmed that SHP-1 interacted with VEGFR2 following TNF-α stimulation, reducing the proliferation of primary EC (64, 65) while silencing SHP-1 increased the activation of VEGFR2 pathway resulting in improved VEGF-induced EC proliferation and capillary formation (66, 67). In the meantime, Sugano et al. was the first to report that inhibition of ischemia-induced SHP-1 expression markedly increased VEGFR2 activation and capillary density in the HLI rat model (68).

The influence of SHP-1 in diabetic collateral vessel angiogenesis has been partially brought to light. Using the HLI model, a first study uncovered that hyperglycemia-driven PKC-δ activation increased both mRNA and protein expression of SHP-1 in the muscle of STZ-induced T1D mice 28 days post-ligation of the femoral artery. The upregulation of SHP-1 was accompanied by a decrease in both VEGFR2 and PDGFR-β signaling, leading to reduced capillary density and blood flow reperfusion. Whole-body deletion of PKC-δ re-established the angiogenic pathways in the diabetic ischemic limb by suppressing SHP-1 overexpression (41). In the same model, our group later reported that the deletion of AT2 receptor suppressed diabetes-induced binding of SHP-1 to VEGFR2, therefore restoring its signaling to improve blood flow reperfusion following 28 days of HLI (42). Interestingly, the exposure of BAEC to high glucose had no effect on SHP-1 expression but increased its phosphatase activity and direct interaction with VEGFR2 through the activation of AT2R, resulting in decreased EC migration (42). Finally, Chen et al. evaluated the effect of hyperglycemia on SHP-1 interaction with the Ang-1/Tie2 pathway in MI. SHP-1 was upregulated in the heart of T2D mice which was associated with blunted vessel outgrowth and capillary density. Administration of a broad-spectrum PTP inhibitor suppressed SHP-1 expression and improved Tie2 signaling. Further cell-based assays uncovered that high glucose levels increased SHP-1/Tie2 interaction and reduced Tie2 phosphorylation by decreasing Ang-1-mediated SHP-1/Tie2 dissociation in EC (48).

### Src Homology Region 2 Domain-Containing Phosphatase-2

Src homology 2 domain-containing phosphatase 2 (SHP-2, gene: *PTPN11*) is a ubiquitously expressed non-transmembrane phosphatase sharing a similar structure and phosphatase activity mechanism as SHP-1. Unlike SHP-1, the global deletion of SHP-2 is embryonically lethal (107). SHP-2 have been shown to interact with several pro-angiogenic RTK including PDGFR-β (108), VEGFR2 (106) and Tie2 (109). In contrast to the exclusively negative role of SHP-1, SHP-2 exerts a dual regulatory role on RTK. Overall, SHP-2 has been widely described as a positive regulator of angiogenesis through the activation of the Ras/ERK pathway (87). However, silencing SHP-2 or inhibiting its catalytic domain increased ERK activation following VEGF-A stimulation which contradicts its role as a positive regulator (110). In addition, SHP-2 has been shown to directly bind to the Y1175 residue and dephosphorylate VEGFR2 upon VEGF-A stimulation, therefore reducing aortic EC migration (111). Conversely, overexpressing an inactive form or SHP-2 knockdown restored VEGFR2 activation and angiogenic properties of EC (69, 111). Moreover, SHP-2 is a positive regulator of the PI3K/Akt axis. Hence, inhibition of SHP-2 decreased the activation of Src and downstream Akt/eNOS effectors in response to VEGF-A in HUVEC through hyperactivity of the C-terminal SRC kinase (Csk) (110). SHP-2 was also critically required to fully activate Src/Akt in EC and subsequently induce migration and capillary formation following association with the docking adaptor Gab1 (70). SHP-2 has also been shown to bind, dephosphorylate and ultimately inhibit Tie2 signaling (92).

Despite its dual role, little is known about the regulation of SHP-2 in PAD. Indeed, SHP-2 levels remained unchanged in the ischemic hindlimb muscle of rats 3 weeks post excision of the femoral artery (68). Similarly, no variations in mRNA and protein levels of SHP-2 were observed in the ischemic muscle of T1D mice compared to ischemic controls, suggesting that SHP-2 might have a lesser impact than SHP-1 in collateral vessel formation in diabetic PAD (41, 42). However, a recent study reported that MGO-exposed human monocytes or monocytes collected from T2D patients and *db*/*db* T2D mice showed excessive and abnormal migration through enhanced activity of SHP-2, leading to dephosphorylation of Src and consequent paxillin activation (98). This suggests that hyperglycemia-induced upregulation of SHP-2 could have a critical role in monocyte dysfunction by promoting VEGF resistance to ultimately alter arteriogenesis in diabetic PAD patients.

### Vascular Endothelial PTP

Vascular endothelial PTP (VE-PTP, gene: *PTPRB*) belongs to the receptor PTP (PTPR) family anchored at the cell membrane. VE-PTP is exclusively expressed in EC (92). *Ptprb*^−−/−^ mouse embryos die between E9.5-10 due to severe disorganization of the primary vascular plexus and lack of functional blood vessels (112, 113). Due to its predominant expression in EC, it is not surprising that VEGFR2 is the main target of VE-PTP (114). Indeed, upon VEGF-A stimulation, VE-PTP was shown to bind and quickly dephosphorylate VEGFR2. Silencing VE-PTP with an siRNA further increased the degree of VEGF-A-induced VEGFR2 phosphorylation and signaling. This lead to an increase in EC proliferation but at the expense of tubule formation (114). Interestingly, VE-PTP required the recruitment of Tie2 to form a complex and dephosphorylate VEGFR2 in EC. The deletion of VE-PTP induced hyperproliferation of stalk cells following VEGF-A stimulation and caused an excessive sprouting of non-functional lumen-free neo-vessels (71). Interestingly, Tie2 was previously found to be an independent direct target of VE-PTP in mice EC (115). Ang-1 stimulation increased the binding of VE-PTP with Tie2 in HUVEC. In addition, silencing VE-PTP enhanced Tie2 signaling, ERK activation, and EC over-proliferation, ultimately triggering pathological vessel enlargement in juvenile mice (72). Taken together these studies uncovered VE-PTP as a physiological molecular brake of VEGFR2/Tie2 pro-angiogenic pathways favoring vessel maturation.

VE-PTP has been poorly investigated in PAD and only an unchanged expression was found in the diabetic ischemic muscle of STZ-induced T1D mice 28-days post ligation of the femoral artery (42). Nevertheless, a recent investigation showed that VE-PTP was upregulated in the renal microvasculature, which resulted in reduced Tie2 activation, increased neo-vessel destabilization and ultimately the progression of diabetic nephropathy (73). Similarly, local inhibition of VE-PTP promoted Tie2 activation, reduced vascular leakage, and neovascularization in diabetic macular edema (116). Although VE-PTP was viewed as a potent player in the progression of diabetic microvascular complications through the deregulation of VEGFR/Tie2 angiogenic pathways, its role in lower limb angiogenesis still need to be clarified.

### Density Enhanced Phosphatase 1

Density enhanced phosphatase 1 (DEP-1, gene: *PTPRJ*) is the second member of the PTPR family and shares mechanistic analogies with VE-PTP. Nevertheless, since DEP-1 expression is not restricted to EC, it can bind a larger range of pro-angiogenic RTK mainly VEGFR2 (117) and PDGFR-β (76). Hence, DEP-1 directly bound and selectively dephosphorylated the latter upon PDGF-BB stimulation, which in turn negatively affected PDGFR signaling in EC and fibroblasts (118) as well as in VSMC (74). Similar findings were made regarding VEGF signaling since DEP-1 bound (117) and dephosphorylated VEGFR2 at multiple sites following VEGF-A stimulation in HUVEC (119) leading to blunted VEGFR2/ERK axis and EC angiogenic properties (75). In contrast, DEP-1 exerted an indirect positive regulation of VEGFR2 by dephosphorylating inhibitory Y529 residue leading to enhanced EC angiogenic properties and permeability (77).

Although molecular mechanisms of DEP-1 are well-establish *in vitro*, little is known about its role in ischemia-induced hindlimb angiogenesis in PAD. Using the HLI model, a first study failed to detect variations in blood reperfusion or vessel generation in *Ptprj*^−/−^ mice 7 days post-surgery (94). The deletion of DEP-1 reduced limb reperfusion and capillary density in HLI mice 14 days post-surgery compared to controls, which correlated with a loss of mobility and an increased amount of necrotic fingers (95). These data suggest that, despite a prominent negative regulatory role of pro-angiogenic RTK *in vitro*, DEP-1 might be critically required in lower limb collateral angiogenesis *in vivo* through the positive regulation of pro-angiogenic RTK downstream effectors. Nevertheless, the impact of diabetes on the regulation of DEP-1 and further angiogenic outcomes still need to be evaluated in both *in vitro* and *in vivo* preclinical models.

### T-Cell Protein Tyrosine Phosphatase

T-cell PTP (TC-PTP, gene: *PTPN2*) is an intracellular ubiquitously expressed non-receptor PTP. TC-PTP was found to be expressed in both EC and VSMC (92) as well as involved in the regulation of VEGFR2 (120), PDGFR-β (108) but also epithelial growth factor receptor (EGFR) signaling (79). Using site-selective antibodies, Persson et al. showed an increase in the auto-phosphorylation of four PDGFR-β tyrosine residues following PDGF-BB stimulation in TC-PTP KO mice fibroblasts compared to control mice. The deletion of TC-PTP raised the phosphorylation of PDGFR-β at Y1021, which led to increase PLCγ activation and subsequent fibroblast migration (121). Besides PDGFR-β, TC-PTP co-precipitated with VEGFR2 upon VEGF-A stimulation in EC and selectively dephosphorylated several residues but not Y1175. Consequently, VEGF-A-driven HUVEC proliferation was modestly improved by silencing TC-PTP whereas migration and sprout formation were increased (120, 122).

The regulation of TC-PTP expression or activity has never been explored in preclinical models of diabetic PAD and the associated angiogenic outcomes are currently unknown. However, a recent study has reported a decrease in TC-PTP expression in the renal cortex of T1D mice, which was associated with increased fibrosis and progression of diabetic nephropathy suggesting a protective role of TC-PTP (78). Unlike promoting excessive angiogenesis in diabetic nephropathy, TC-PTP could be likely upregulated in the context of diabetic PAD leading to VEGF unresponsiveness. Nonetheless, this assumption must be assessed with further experiments using diabetic HLI models and cultured EC exposed to high glucose levels.

### Phosphatase and Tensin Homolog

Phosphatase and tensin homolog (PTEN, gene: *PTEN*) is a ubiquitous lipid and protein phosphatase. PTEN is also the main regulator of the PI3K/Akt pathway by dephosphorylating phosphatidylinositol-3,4,5-trisphosphate (PIP3) into phosphatidylinositol-3,4-bisphosphate (PIP2), therefore indirectly preventing RTK-induced Akt activation (80). A mechanistic study reported that the overexpression of wild-type PTEN dampened VEGF-A-induced Akt activation and subsequent proliferation, migration, and tubule formation in HUVEC. Furthermore, overexpressing a dominant-negative form of PTEN rescued the Akt pathway and EC angiogenic properties (123). In a similar study, overexpressing PTEN inhibited the PDGF-BB-induced FAK/Akt/p70S6K cascade reducing proliferation and migration of VSMC (124). Interestingly, PTEN expression was upregulated by NF-κB in hypoxic EC and normalized following Ang-1 treatment (81).

PTEN was also upregulated in both aortic homogenates from T1D mice and EC exposed to high glucose concentrations which was associated with increased apoptosis. On the other hand, silencing PTEN restored Akt activation and EC viability similar to normoglycemic exposed cells (82). The upregulation of PTEN in HG-exposed EC and aorta of STZ-induced diabetic mice was dependent on endothelial upregulation of p53 (99).

Although the above-mentioned studies demonstrate that the anti-angiogenic role of PTEN could be potentiated by both ischemia and hyperglycemia, no data are currently available in the setting of diabetic PAD. Nevertheless, a clinical investigation revealed significantly increased expression of PTEN along with a decreasing trend in the activation of Akt/eNOS in atrial biopsies of T2D patients compared to healthy individuals. Consequently, this raises the assumption that similar PTEN activity may occur in ischemic diabetic limbs (125).

### Dual-Specificity Phosphatases

Dual specificity protein phosphatases (DUSPs) are either cytoplasmic or nuclear phosphatases acting upon tyrosine or serine/threonine residues which are responsible for dephosphorylation and regulation of MAPKs (ERK, p38, and JNK). Therefore, DUSPs indirectly participate in the regulation of RTK-induced angiogenesis. Several studies reported a pro-angiogenetic role of multiple DUSPs. For instance, silencing DUSP1 blunted VEGF-A-induced migration (83, 96), proliferation (85), and tubule formation in HUVEC (96) as well as *ex vivo* EC sprouting (96). Fibroblasts isolated from DUSP4 KO mice exhibited reduced proliferation and increased apoptosis due to a sustained overactivation of ERK and JNK, respectively (86). Conversely, overexpressing DUSP1 and DUSP5 abolished p38/ERK activation and subsequent HUVEC migration/proliferation suggesting that a proper balance in DUSPs expression is critical for maintaining angiogenic properties of EC (85). High glucose exposure was shown to downregulate DUSP1 expression in VSMC causing increased and sustained activation of p38/ERK which was associated with excessive proliferation of VSMC (100). Recently, DUSP5 expression was found to be increased in hypoxic HUVEC but not in VSMC (84).

In the HLI model, the deletion of DUSP1 led to a significant reduction in blood flow reperfusion at days 3 and 7 post-surgery, gradually recovering to levels similar to controls by day 28. Muscle vessel density was altered in the same time frame highlighting the critical role of DUSP1 in the early response to hindlimb ischemia (96). Knocking-down DUSP5 also significantly reduced perfusion recovery and was correlated with an increase in limb necrosis which persisted 4 weeks post-surgery (84). Unfortunately, no studies have yet been conducted in the setting of diabetic PAD. Nevertheless, since diabetes-induced downregulation of DUSP1 (126) and DUSP4 (89) have been associated with worsened diabetic nephropathy, it is likely that any variations in DUSPs expression or activity could also contribute to the alteration of collateral vessel formation in the context of diabetic PAD.

## Concluding Remarks and Future Perspectives

**Figure 4:**
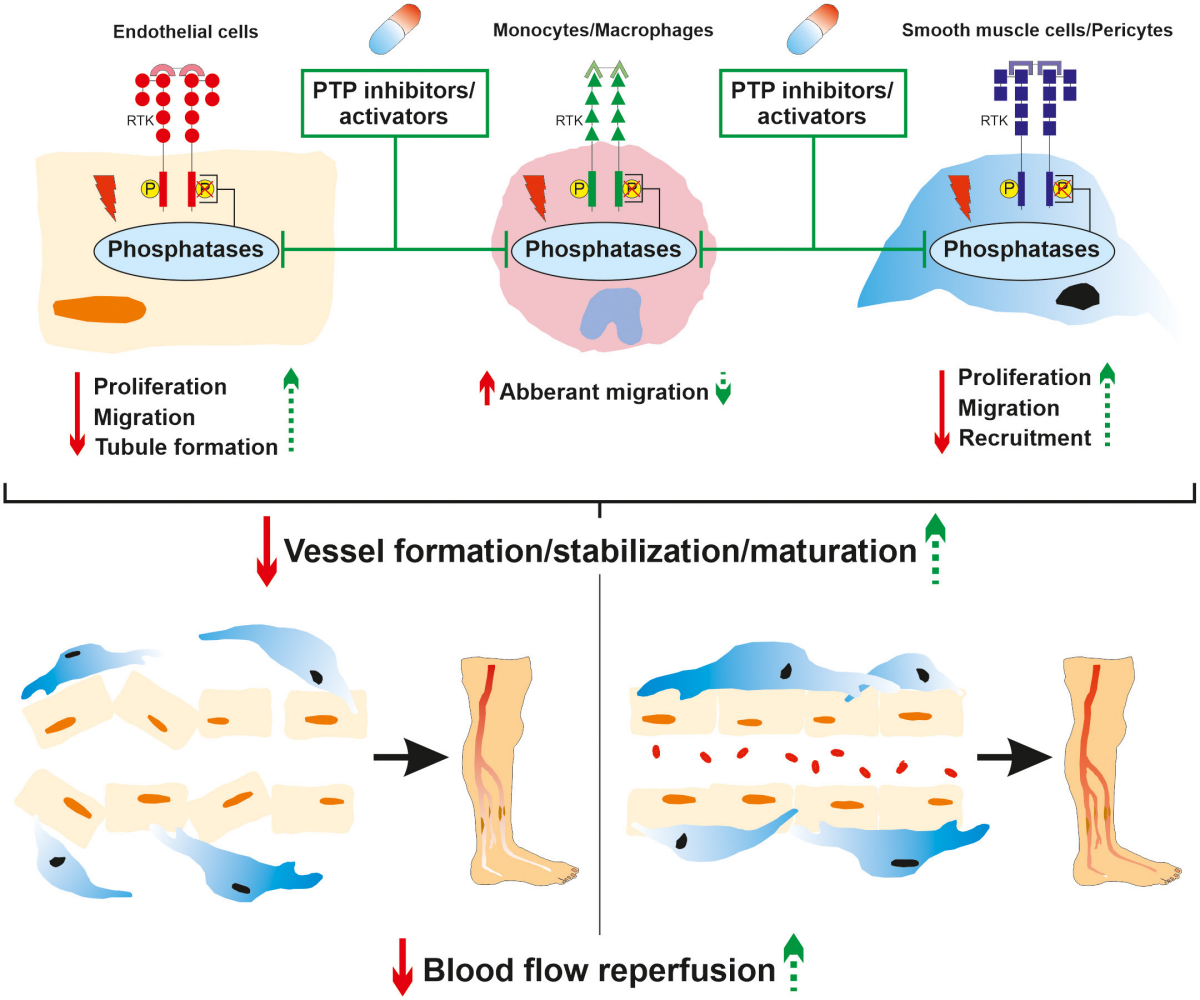
Role of phosphatases in diabetic PAD and therapeutic potential of targeted PTP drugs. Hyperglycemia-induced modulation of phosphatase expression or activity contributes to pro-angiogenic receptor tyrosine kinase signaling deregulation. This led to dysfunctions in endothelial cell, macrophage/monocyte and VSMC/pericyte angiogenic properties that subsequently impaired proper neo-vessel formation resulting in insufficient angiogenesis in the ischemic lower limb. The local administration of both PTP and cell specific inhibitors/activators could partially restore endothelial, immune, and perivascular cell angiogenic properties. This could contribute to improve neo-vessel generation, maturation, and stabilization to ultimately enhance blood flow reperfusion in the ischemic lower limb of diabetic PAD patients.

Hyperglycemia is deeply involved in the progression of macrovascular complications by deregulating cellular responses to hypoxia. Diabetic PAD is characterized by insufficient angiogenesis of the lower extremities which is attributed to impaired production of pro-angiogenic factors and defects in signal transduction in both vascular and perivascular cells. Although diabetes-induced alteration of VEGF signaling is extensively proven, there is still a lack of conclusive large-scale clinical studies concerning other growth factors such as PDGF and angiopoietins. Hence, larger clinical studies supported by suitable preclinical mechanistic models are required to guide and optimize the development of new therapeutic targets based on the administration of exogenous growth factors which are currently lacking sustainable effects. The source of vascular complications in diabetes is tremendously multifactorial and PTP have progressively emerged as potent players in vascular cell unresponsiveness to growth factors. Although the role of PTP in the modulation of pro-angiogenic RTK signaling is well-documented at a physiological level, their regulation in the complex hypoxic and hyperglycemic environment in PAD remains partially understood. Because the expression of PTP is not strictly limited to the endothelial lineage but extended to perivascular cells, the use of simultaneous cell and phosphatase-specific knockout models represent the next step in improving knowledge on this subject. In addition, since diabetic PAD is in part characterized by vascular and perivascular cell unresponsiveness to pro-angiogenic growth factors due to the upregulation or downregulation of PTP activity, the development of PTP inhibitors and activators is also an exciting avenue to modulate pro-angiogenic RTK activity (Figure 4). From a pharmacological point of view, the development of such drugs should ideally meet three criteria: PTP selectivity, cell population specificity, and ability to be locally delivered. PTP selectivity is crucial since not all PTP are exclusively negative regulators of pro-angiogenic RTK. Some PTP are critically required for proper angiogenic signal transduction through the dephosphorylation of inhibitory residues in downstream effectors (e.g., Y527 inhibitory on Src). The importance of cell population specificity could be found in the tight balance between angiogenesis and atherogenesis. Hence, the inhibition of PTP (e.g., SHP-1) in EC could promote enhanced VEGFR2 activation to improve EC angiogenic properties and the subsequent formation of neo-vessels. However, simultaneous overactivation of PDGFR-β through the inhibition of SHP-1 in VSMC may lead to uncontrolled proliferation and migration, representing a hallmark of restenosis. Finally, local delivery of PTP drugs exclusively in the ischemic muscle of PAD patients is desirable as pathological events can arise from the modulation of these ubiquitous proteins in other body locations. In a context of vascular complications, the systemic inhibition of PTP could trigger excessive angiogenesis in non-targeted tissues leading to the progression and worsening of diabetic retinopathy (127) or nephropathy (128). PTP drug-induced exacerbated and uncontrolled angiogenesis should also be avoided in the ischemic limb as it can promote atherosclerosis and plaque instability to subsequently increase the risk of PAD (129, 130). Local growth factor inhibitors such as anti-VEGF (131) or anti-Ang-2 (132) antibodies could be used along with PTP drugs to slow down the formation of atherosclerotic lesions and stabilize the neo-vasculature.

## Data Availability Statement

The original contributions presented in the study are included in the article/supplementary materials, further inquiries can be directed to the corresponding author/s.

## Author Contributions

CM and PG conceived the idea for the review. CM performed the literature search and wrote the manuscript. PG and MR critically revised the work. All authors contributed to the article and approved the submitted version.

## Conflict of Interest

The authors declare that the research was conducted in the absence of any commercial or financial relationships that could be construed as a potential conflict of interest.

## Abbreviations

Akt: protein kinase B
CLI: critical limb ischemia
CVD: cardiovascular disease
DUSP: dual-specificity phosphatase
EC: endothelial cell
eNOS: endothelial nitric oxide synthase
EPC: endothelial progenitor cell
ERK: extracellular signal-regulated kinase
PAD: peripheral artery disease
PDGF: platelet-derived growth factor
PDGFR: platelet-derived growth factor receptor
PTP: protein tyrosin phosphatase
RTK: receptor tyrosine kinase
SDF-1: stromal cell-derived factor 1
STZ: streptozotocin
sVEGFR: soluble vascular endothelial growth factor receptor
T1D: Type I diabetes
T2D: Type II diabetes
VEGF: vascular endothelial growth factor
VEGFR: vascular endothelial growth factor receptor
VSMC: vascular smooth muscle cells
PTP1B: protein tyrosine phosphatase 1B
VE-PTP: Vascular endothelial PTP
PTEN: Phosphatase and tensin homolog
HbA1c: glycated hemoglobin
HLI: Hindlimb ischemia
PKC: protein kinase C
AT2R: angiotensin type 2 receptor
Ang-1/2: angiopoietin 1/2
SHP-1/2: Src homology region 2 domain-containing phosphatase-1/2.
